# Macromolecular crystallography at Elettra: current and future perspectives

**DOI:** 10.1107/S1600577525001055

**Published:** 2025-03-26

**Authors:** Raghurama P. Hegde, Nicola Demitri, Annie Héroux, Alessandro Olivo, Giorgio Bais, Michele Cianci, Paola Storici, Dan-George Dumitrescu, Nishant Kumar Varshney, Balasubramanian Gopal, D. D. Sarma, Lisa Vaccari, Silvia Onesti, Maurizio Polentarutti

**Affiliations:** a Elettra – Sincrotrone Trieste SCpA SS 14 km 163,5 in AREA Science Park Basovizza 34149Trieste Italy; b Former Elettra – Sincrotrone Trieste SCpA SS 14 km 163,5 in AREA Science Park Basovizza 34149Trieste Italy; c Department of Agricultural, Food and Environmental Sciences, Università Politecnica delle Marche, via Brecce Bianche 10, 60131Ancona, Italy; d Nobian, Van Asch v. Wijckstraat 53, 3811 LPAmersfoort, The Netherlands; e IR Technology Services Pvt Ltd, EL-91, TTC Industrial Area, Electronic Zone, Mahape, Navi Mumbai, Maharashtra400710, India; f Molecular Biophysics Unit, Division of Biological Sciences, Indian Institute of Science, Bengaluru560012, India; g Solid State and Structural Chemistry Unit, Indian Institute of Science, Bengaluru560012, India; Cornell University, USA

**Keywords:** data collection, X-ray diffraction, macromolecular crystallography, Elettra upgrade

## Abstract

The Elettra synchrotron radiation facility, located in Trieste, Italy, is a third-generation storage ring, operating in top-up mode at both 2.0 and 2.4 GeV. The facility currently hosts one beamline fully dedicated to macromolecular crystallography, XRD2. XRD2 is based on a superconducting wiggler, and it has been open to users since 2018. On-site and remote access for data collection, as well as monitoring tools and automatic data analysis pipelines are available to its users. In addition, since 1994 Elettra has operated a general-purpose diffraction beamline, XRD1, offering the macromolecular community a wide spectrum extending to long wavelengths for phasing and ion identification. Ancillary facilities support the beamlines, providing sample preparation and a high-throughput crystallization platform for the user community. A new CryoEM facility is being established on campus and jointly operated by the Consiglio Nazionale della Ricerche – Istituto Officina dei Materiali (CNR–IOM) and Elettra, providing further opportunities to the Elettra user community. This review outlines the current capabilities and anticipated developments for macromolecular crystallography at Elettra to accompany the upcoming upgrade to Elettra 2.0, featuring a six-bend enhanced achromat lattice. The new source is expected to deliver a high-brilliance beam, enabling the macromolecular crystallography community to better address the emerging and future scientific challenges.

## Abstract 2

The XRD2 beamline at Elettra-Sincrotrone Trieste has been in operation since 2018 and is dedicated to macromolecular crystallography for both academic and industrial research, a role partially fulfilled, before 2018, by XRD1, a general-purpose diffraction beamline. With the upcoming Elettra 2.0 upgrade, based on a six-bend enhanced achromat lattice, the synchrotron will offer a brighter, more powerful beam to address evolving challenges in crystallography.

## 1. Introduction

Elettra-Sincrotrone Trieste (henceforth referred to as Elettra) is an Italian non-profit organization of national interest that oversees a multidisciplinary research centre of excellence. Its third-generation synchrotron source, Elettra, has been operational since 1993. Elettra currently powers 28 experimental stations, providing access to cutting-edge instruments for advanced spectroscopy, scattering and imaging techniques. Elettra has consistently enhanced its performance through regular upgrades to its ring, its beamlines, and its experimental stations.

Elettra is currently the only synchrotron radiation facility that regularly operates at two distinct energy levels, 2.4 and 2.0 GeV, depending on users’ requirements for hard X-rays or VUV photons. Additionally, it offers a hybrid mode for time-resolved experiments. Out of its 28 beamlines, 9 are dedicated to hard X-rays, 18 operate within the UV, soft, and tender X-ray range, and 1 focuses on the IR-THz range.

A number of beamlines have been built and are being operated in collaboration with national and international institutions and research centres. Nine beamlines are managed by the Italian National Research Council (CNR), including the X-ray diffraction beamline XRD1. Several international institutions have established beamlines at Elettra. The SAXS beamline was built by the Austrian Academy of Sciences and currently run together with the Institute of Inorganic Chemistry (Graz University of Technology). The Materials Science beamline was built and is run by Charles University in Prague, and the macromolecular crystallography XRD2 and the high pressure diffraction XPRESS beamlines were built and now run in partnership with the Indian scientific community via the Department of Science and Technology and Indian Institute of Science in Bangalore. The NanoESCA, SuperESCA and XRF beamlines are operated in partnership with institutions from Germany, Romania and the International Atomic Energy Agency (IAEA), respectively.

Approximately 80% of the beam time at Elettra (∼6400 h per year) is allocated to the user community, based on the ranking of user proposals by international peer-review panels (Franciosi & Kiskinova, 2023 ▸). In 2024, over 500 scientists visited Elettra to conduct research spanning a variety of scientific fields. More than half of the users come from non-Italian institutions, with growing participation from Central-Eastern European and developing countries, thanks to ongoing collaborations with the Central European Initiative (CEI), the International Atomic Energy Agency (IAEA), the International Centre for Theoretical Physics (ICTP), the Central European Research Infrastructure Consortium CERIC-ERIC and the Lightsources for Africa, The Americas, Asia, Middle East and Pacific (LAAAMP) exchange programme. In the last five years, research conducted at the facility has resulted in more than 3000 scientific publications.

Elettra is undergoing a major upgrade to Elettra 2.0 (Karantzoulis *et al.*, 2024 ▸; Gregoratti *et al.*, 2024 ▸), a next-generation diffraction-limited light source expected to be operational from January 2027, offering enhanced brilliance and coherence. In this context, the present XRD2 beamline, dedicated to macromolecular crystallography, will be replaced by a new beamline (µXRD). The upgrade integrates with a broader expansion of the Elettra’s structural biology platforms funded by the NextGenerationEU programme, through the PRP@CERIC project (https://www.pathogen-ri.eu). This includes enhanced crystallization and protein characterization facilities, as well as the establishment of a new CryoEM facility, in collaboration with the CNR Istituto Officina dei Materiali.

## 2. Macromolecular crystallography (MX) at Elettra: current status

Prior to 2018 MX data collection was carried out at XRD1, a general-purpose diffraction beamline, that acted as an incubator, fostering multiple scientific communities and opening the way to dedicated lines such as XRD2 for MX and Xpress for high pressure diffraction (Lausi *et al.*, 2015 ▸). The MX dedicated XRD2 beamline has been designed and realized as a partnership between Elettra and the Department of Science and Technology (DST), India/Indian Institute of Science (IISc, Bangalore). From its inception, XRD2 was designed with modern features essential for an MX beamline, focusing on stability and automation capabilities for remote access and operation. Several models were taken into consideration before adopting the final beamline layout and software environment. XRD2 went officially into operation in September 2018.

### 2.1. The macromolecular crystallography XRD2 beamline

**Figure 1:**
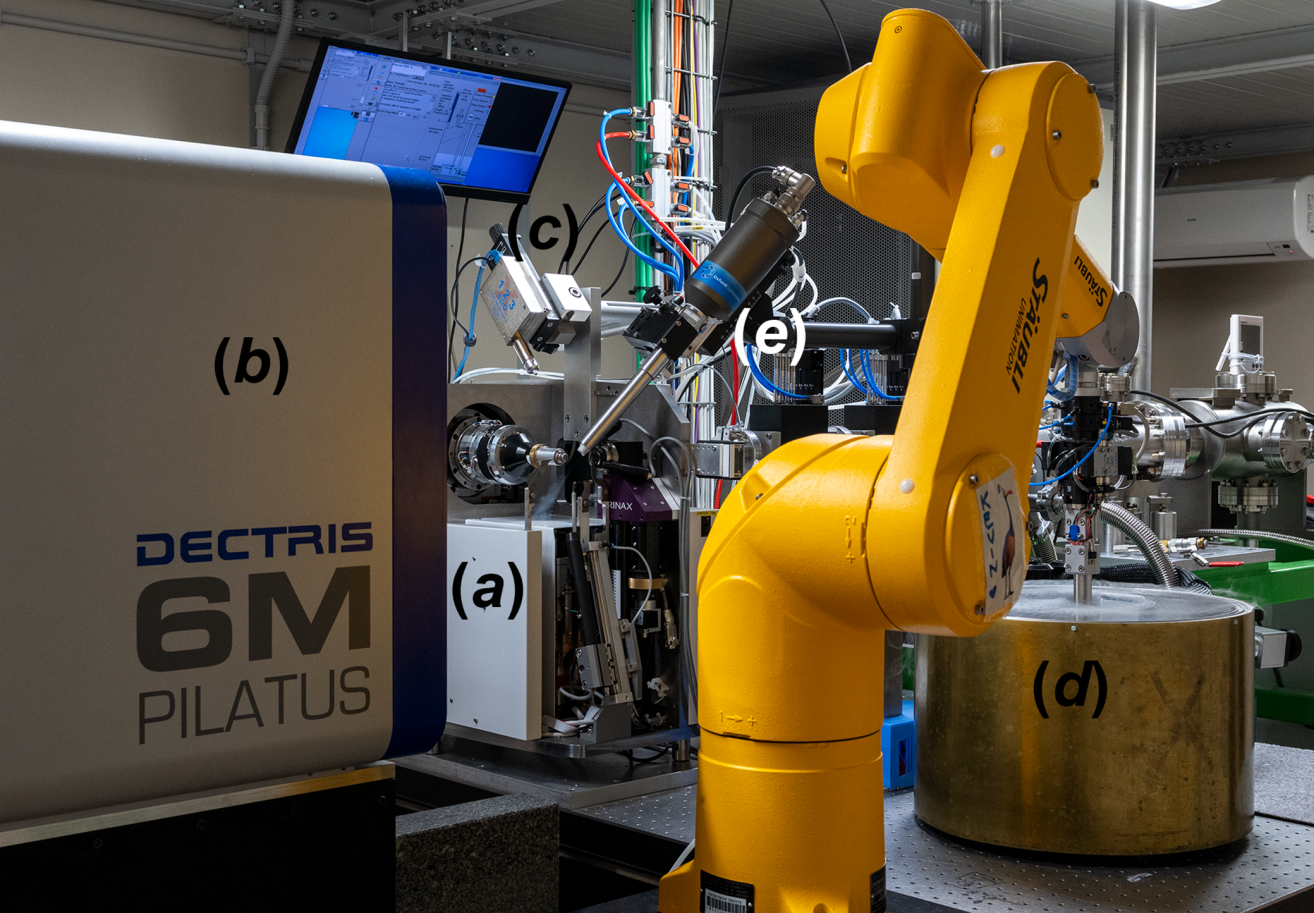
XRD2 experimental station consisting of (*a*) the Arinax MD2-S microdiffractometer, (*b*) Pilatus 6M detector, (*c*) fluorescence detector, (*d*) automounter and (*e*) cryostream.

**Figure 2:**
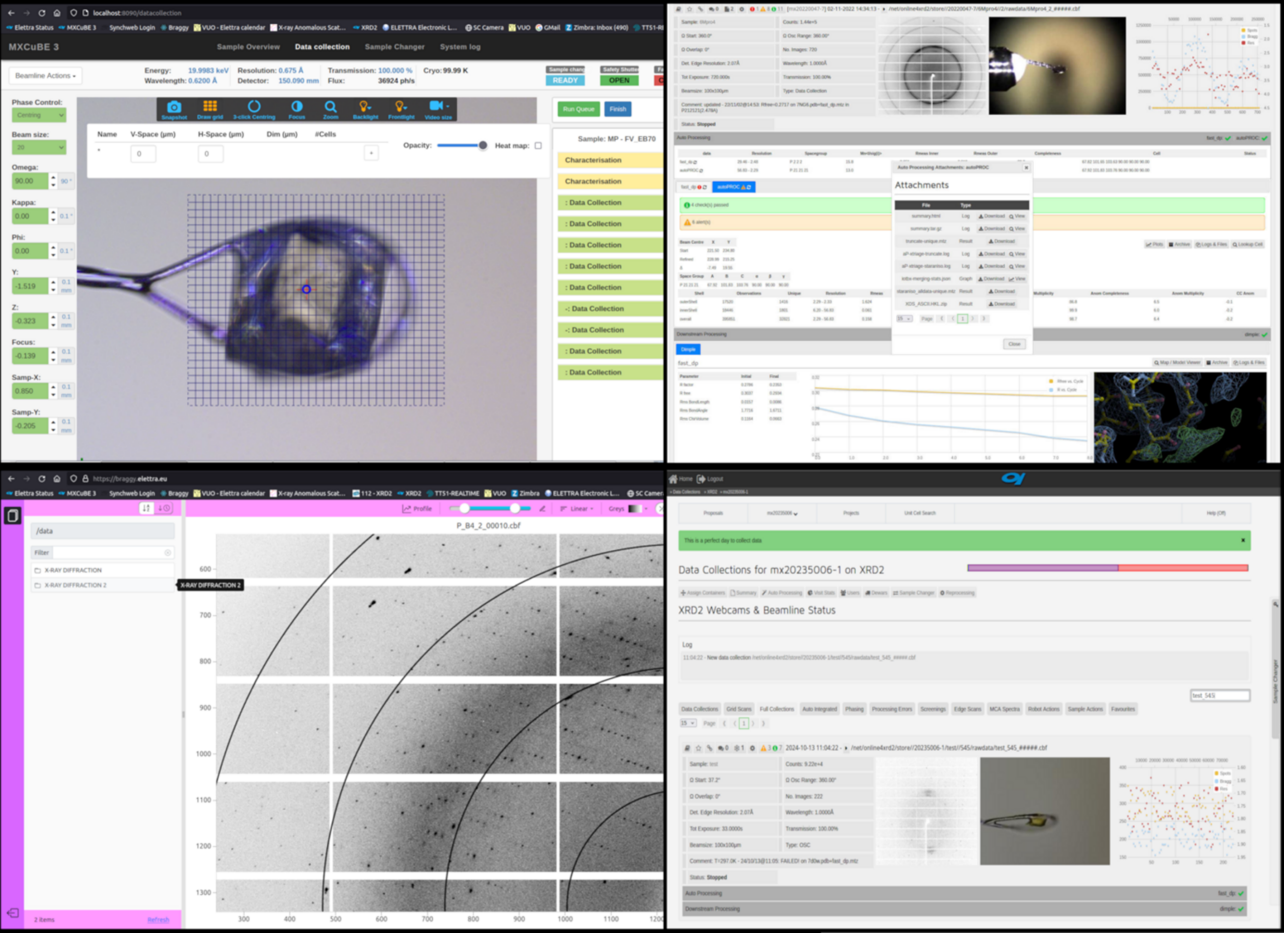
A composite image of the web-based software tools available to remote and in-person users at the XRD2 beamline showing (counterclockwise starting from top-left): *MXCuBE3* beamline control interface, visualization software for diffraction images, *Braggy*, and *SynchWeb/ISPyB* data logbook showing some results from autoprocessing of data (*fastdp/autoPROC/Dimple*).

The source for XRD2 is a superconducting wiggler delivering beam in parallel to three beamlines of which XRD2 is the central branch. The photon-delivery system provides at the sample position a native beam of 290 µm × 90 µm (h × v, FWHM) with a flux of 1.7 × 10^12^ photons s^−1^ (12.4 keV). The beam energy can be tuned between 8 and 20 keV, suitable for single- and multi-wavelength anomalous dispersion (SAD/MAD) phasing. The experimental station (Fig. 1 ▸) is equipped with an automounter system, coupled with a dewar hosting up to 192 samples, an Arinax MD2-S diffractometer, and a Dectris Pilatus 6M detector. Since 2020, most users are performing their experiments remotely, using the web application *MXCuBE3* for beamline control (Mueller *et al.*, 2017 ▸), *Braggy* for diffraction images inspection (https://gitlab.esrf.fr/ui/h5web-braggy) and *SynchWeb/ISPyB* (Fisher *et al.*, 2015 ▸; Delagenière *et al.*, 2011 ▸) as electronic logbook (Fig. 2 ▸).

#### 2.1.1. Beamline source

XRD2 exploits as a source a 3.5 T cryo-cooled (He) superconducting wiggler (SCW, Table 1 ▸) with a large emitting cone limited to 2.0 mrad and 0.2 mrad (horizontal and vertical directions, respectively). The source offers wide, intense spectra extending well beyond 50 keV. The divergence of the radiation cone enables the beam to be split, serving as a source for two additional branch beamlines, each accommodating a radiation cone of 0.5 mrad × 0.2 mrad. The central portion of the beam, spanning 1.0 mrad × 0.2 mrad, is utilized by XRD2. Cryo-cooled silicon (111) crystals intercept the incoming beam and propagate fixed-energy photon beams at 25 keV and 13 keV for the Xpress and the incoming HF-SAXS beamlines, respectively.

#### 2.1.2. Beamline optical layout

**Figure 3:**
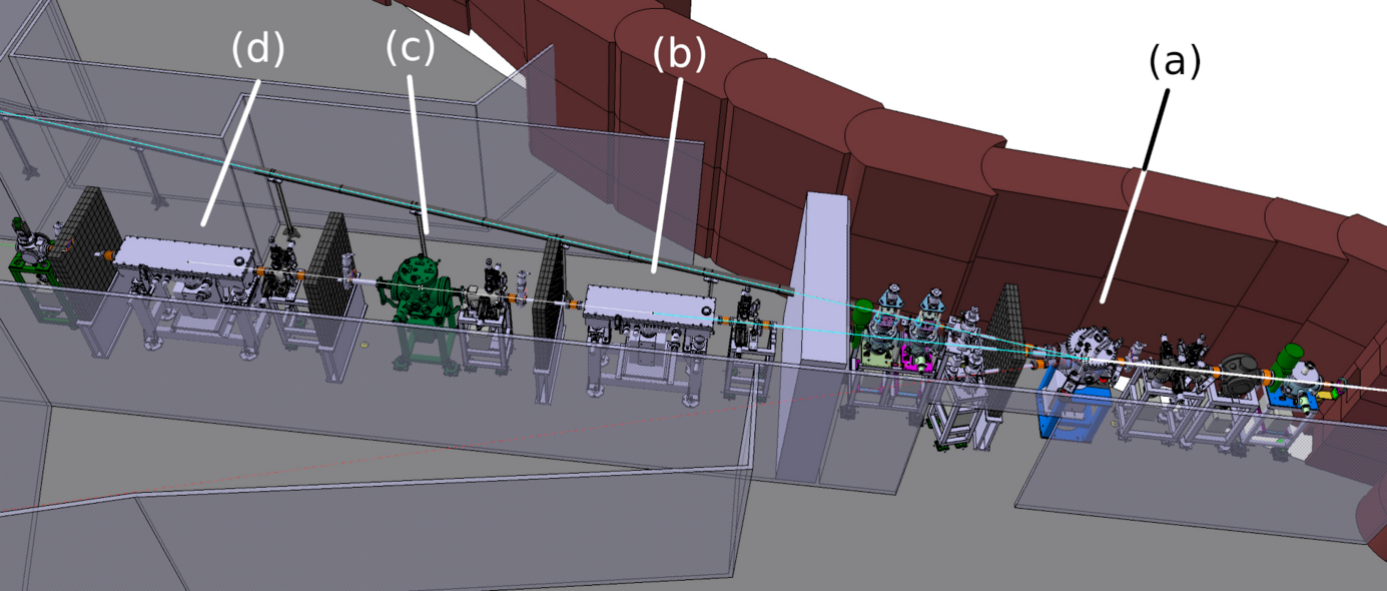
Main elements of the present XRD2 beamline at Elettra. Superconductive source on the right: (*a*) beam splitter, feeding Xpress and HF-SAXS beamlines (the latter not shown) in addition to the central XRD2; (*b*) collimating mirror; (*c*) monochromator and (*d*) focusing mirror.

The main optical components (Table 1 ▸, Fig. 3 ▸) found along the beamline are a platinum-coated vertical collimating mirror and a platinum-coated vertical-bendable toroidal focusing mirror positioned, respectively, before and after a fixed-exit, cryogenic (LN_2_) double-crystal Si(111) monochromator in non-dispersive configuration. The latter is an in-house development (Gambitta, 2010 ▸), with the capability to deliver a monochromatic beam in the range 7.5–36 keV (usually limited to 8–20 keV for MX measurements) with the Si(111) bandwidth resolution. Most of the data collections are performed at wavelengths around 1.0 Å. The beam position and intensity at the experiment are measured using a four-quadrant beam position monitor (Alibava Systems) and stabilized/optimized via a feedback system acting on the optical components.

The measured size of the focal spot and the relative angular divergences confirm the expected values, 290 µm × 90 µm (FWHM) and 2.0 mrad × 0.25 mrad (FWHM), respectively, with a native full flux of 1.7 × 10^12^ photons s^−1^ at 1 Å. With a typical aperture of 100 µm the flux would be ∼6 × 10^11^ photons s^−1^. A typical data collection at XRD2 on a 100 µm × 100 µm × 100 µm crystal would result in an average diffraction weighted dose of 5.6 MGy, as calculated using *RADDOSE-3D* software (Zeldin *et al.*, 2013 ▸), which is within the Garman limit (Owen *et al.*, 2006 ▸).

#### 2.1.3. Experimental station

The main components of the end-station are an MD2-S diffractometer (Arinax) combined with a large-area Pilatus 6M detector (Dectris) with a sensor thickness of 320 mm and a maximum readout rate of 12 Hz (Fig. 1 ▸). A set of round apertures of sizes 20, 50, 75, 100 and 150 µm allows one to adapt the size of the beam to the samples. Apertures are followed by a clean-up capillary extending to the crystalline sample to reduce the image background. A set of attenuators can be used to reduce the beam intensity if the sample(s) are expected to undergo radiation damage. The standard acquisition time is about 1 s per degree or 6 min for a full sphere with unattenuated beam and aperture of 100 µm, allowing daily data collections of samples contained in ten pucks.

Ancillary equipment includes a fluorescence detector (X123 SDD from Amptek) used for beamline calibration, for very rare MAD measurements or for the identification of an (unexpected) bound heavy atom. The setup allows also one to anneal the sample to enhance its crystalline order via the control interface. The automounter offers a storage dewar hosting up to 192 SPINE-compliant samples based on UNIPUCKS-type pucks. Its open-lid configuration provides quick access and easy insertion and removal of pucks. The integration between the robotic arm (Stäubli TXL-60), dewar and diffractometer was developed in-house. Automatic reading/checking of the pin data matrix is available and is encouraged. The IRELEC double gripper minimizes the duty cycle between samples to ∼40 s, which remains negligible compared with typical data collection times, and allows a full shipping canister screening in less than 4 h.

### 2.2. The general purpose X-ray diffraction XRD1 beamline

The X-Ray Diffraction 1 (XRD1) beamline has been designed – in collaboration with the Istituto di Cristallografia, CNR – to perform a wide variety of measurements and experiments, all based on diffraction (Lausi *et al.*, 2015 ▸). The beamline exploits a 4.5 m wiggler as source of radiation and is equipped with a collimating mirror and a toroidal focusing one, together with a cryogenically cooled (LN_2_), Si(111) double-crystal, fixed-exit monochromator. The latter provides a monochromatic beam in the wide energy range of 4–21.5 keV.

The flux at the sample is in the range 10^12^ photons s^−1^, with a natural beam footprint of 700 µm (H) × 200 µm (V) reduced via a set of round apertures of sizes 200, 100, 50, 20 and 5 µm. Key equipment includes a hybrid pixel area detector (Pilatus 2M from Dectris) and a versatile setup including a κ-geometry goniostat and a robotic arm for handling different samples, hosting single crystals, powders, and thin films in fields ranging from macromolecular crystallography to materials science, under normal, extreme, and special conditions (Polentarutti *et al.*, 2004 ▸).

The availability of longer wavelengths allows us to offer to the MX community the opportunity to exploit the enhancement of anomalous signal from biologically relevant elements such as sulfur, phospho­rus, chlorine and calcium (Mueller-Dieckmann *et al.*, 2004 ▸; Weiss *et al.*, 2004 ▸; Djinovic Carugo *et al.*, 2005 ▸; Mueller-Dieckmann *et al.*, 2005 ▸). Thus, for MX, XRD1 complements XRD2 data collection at longer wavelengths, upon user request, with the option to use a helium purged beam path to mitigate the effects of air scatter (Polentarutti*et al.*, 2004 ▸).

## 3. XRD2 and XRD1 software, computing and data policy

Macromolecular crystallography is a mature field where facilities have invested in dedicated and standardized beamlines while dedicated software, such as *autoPROC* (Vonrhein *et al.*, 2011 ▸), *XDS* (Kabsch, 2010 ▸), *CCP4* (Agirre *et al.*, 2023 ▸) and so on, have streamlined the ever growing needs for guided data collection and fast data analysis. At Elettra, low-level controls are based on the TANGO system control framework (https://www.tango-controls.org/), combined with Python scripting to handle complex functions or build up control interfaces. High-level web interfaces dedicated to data collection, both local and remote, like *MXCuBE3* (Mueller *et al.*, 2017 ▸) and *Braggy* (ESRF, https://gitlab.esrf.fr/ui/h5web-braggy), have been installed and further developed together with the MX software community. They have been made available to MX users, together with automatic pipelines for online data analysis and re-analysis (the latter at the moment on demand only). To help users to make faster and better decisions, *DISTL* (Zhang *et al.*, 2006 ▸) is run as the data collection is progressing to provide a preliminary evaluation of data, *autoPROC* (Vonrhein *et al.*, 2011 ▸) and *fastDP* (Winter & McAuley, 2011 ▸) provide quick metrics for space group, symmetry and results of integration of the data, while *DIMPLE* (https://ccp4.github.io/dimple/) generates electron density maps with focus on interesting electron density blobs. Data analysis is performed on the Elettra High Performance Computing Cluster. Typical data analysis times are comparable with standard data collection durations, enabling users to incorporate prior sample characterization effectively into their decision-making process. MX data and metadata are archived via *ISPyB* (Delagenière *et al.*, 2011 ▸), paired with the *SynchWeb* frontend (Fisher *et al.*, 2015 ▸) and integrated within Elettra’s user portal.

Elettra’s data policy adheres to the FAIR principles — Findability, Accessibility, Interoperability and Reusability — ensuring that all research data generated at the facility are organized, accessible and useful for the scientific community. This policy mandates that all data are stored with comprehensive metadata, facilitating easy discovery and retrieval. By implementing standardized formats, Elettra promotes interoperability, allowing data integration across diverse scientific disciplines. Additionally, data are shared under clear usage terms, fostering reuse while protecting intellectual property rights. Through this FAIR-compliant approach, Elettra supports open science, advancing innovation and collaboration across global research communities.

## 4. Access mode to MX beamlines

Elettra adopted a monthly based proposal submission system to accommodate the needs for fast access to XRD2. All new proposals for individual research groups are sent to the review panel upon submission, and time is allocated at the beginning of the following month. This creates an agile scheduling which fills up the calendar only a week or so in advance by scheduling users when they are ready and eager to collect on freshly produced samples. The scheduling is confirmed when the dewar arrives and easy rescheduling can be done if some delays occur during shipping. Multiple visits can be associated with each proposal, and the scheduling windows are by the hour rather than the usual 8 h shifts.

In order to gain access to *MXCuBE*, the proposal has to be scheduled via the Elettra Virtual Unified Office portal (VUO, https://vuo.elettra.eu), so that users associated with a specific proposal can connect and collect/analyse data using their own VUO credentials.

## 5. Associated facilities for structural biology

A Structural Biology laboratory (SBlab) was established at Elettra with the aim of supporting the MX beamlines and to carry out in-house research, with projects including cancer biology, neurodegenerative diseases, and bacterial and viral infections. In addition to in-house research, SBlab caters to external users via a crystallization and a protein-production facility, which have recently undergone a significant upgrade funded through the NextGenerationEU PRP@CERIC project (https://www.pathogen-ri.eu/).

The protein-production facility is equipped to handle the entire workflow, from gene to protein structure. The crystallization facility includes a MosquitoX3 (TTP-Labtech) equipped with an anti-evaporation chamber, for setting up sitting-drop crystallization trials in 96-well microplates; to support high-throughput monitoring, two Formulatrix Rock Imager 360 systems with UV, VIS and MFI optics have been acquired – one will operate at room temperature and the other at 4°C. These instruments will enable fully automated crystallization processes. The associated software allows users to remotely monitor crystallization experiments in real time.

A new CryoEM facility is being established at the neighbouring CNR Istituto Officina dei Materiali (IOM), and will be jointly managed by IOM and Elettra, providing further structural biology opportunities to the Elettra user community. The facility will include a Glacios2 Cryo-TEM equipped with a Falcon 4 direct electron detector and Selectris energy filter (ThermoFisher Scientific) as well as an Aquilos2 Cryo-FIB/SEM (ThermoFisher Scientific) for the preparation of thin cryo-lamellae. The facility will therefore be able to cover the whole range of electron-based techniques, from single-particle CryoEM analysis, to cryo-electron tomography (CryoET) from biological organelles or frozen cellular lamellas, to electron diffraction on sub-micrometric crystals (MicroED), thus ideally complementing the X-ray diffraction structural biology techniques available at Elettra.

## 6. In-house research

Currently, SBlab hosts the Protein Targets for Drug Discovery group, and the DNA Replication and Repair group. The Protein Targets for Drug Discovery group, investigating structural properties of druggable proteins to support structure-based drug design, was an active player in the concerted effort to explore solutions to treat COVID-19, as participant in the EU EXSCALATE4CoV project (https://www.exscalate4cov.eu/), led by the Italian Pharmaceutical company Dompé, and involving 18 teams from seven EU countries to identify potential compounds binding to viral targets. As a result, 29 structures were deposited in the Protein Data Bank and six publications involved the contribution of XRD2 (Kuzikov *et al.*, 2021 ▸; Pelliccia *et al.*, 2022 ▸; Stefanelli *et al.*, 2023 ▸; Gossen *et al.*, 2021 ▸; Costanzi *et al.*, 2021 ▸; Albani *et al.*, 2024 ▸). This initial effort spun new collaboration opportunities such as a new Horizon Europe project, named AVITHRAPID (Antiviral Therapeutics for Rapid Response Against Pandemic Infectious Diseases, https://avithrapid.eu/).

The DNA Replication and Repair group has long-standing experience in applying molecular and structural biology tools to study the basic genetic processes within the cell, such as DNA replication and repair, using crystallography, electron microscopy, and small-angle X-ray scattering. As genomic instability, which could result from errors in DNA replication and repair, is closely connected with tumour development, this research has been funded over the years by the Italian Association for Cancer Research (AIRC), various INTERREG projects (PROTEO, GLIOMA, TRANS-GLIOMA) and the Marie Curie MSCA network AntiHelix (https://www.elettra.eu/AntiHelix/), aimed at finding inhibitors leading to novel anti-tumour drugs. Most projects focused on the role of helicases in proliferation and genome stability, involving proteins such as the replicative MCM helicase complex and associated factors forming the CMG complex, the PCNA ring (De March *et al.*, 2017 ▸; De March *et al.*, 2018 ▸; Gonzalez-Magaña *et al.*, 2019 ▸), and the FeS helicases DDX11 (Bottega *et al.*, 2021 ▸), FANCJ (Boavida *et al.*, 2024 ▸), RTEL1 (Cortone *et al.*, 2024 ▸), and DinG (De Piante *et al.*, 2023 ▸).

## 7. Highlights from the MX crystallography beamlines

The Elettra MX user community includes several academic and industrial groups from Italy and neighbouring countries, together with a large community of Indian groups supported by the XRD2 Indian partner. The span of their activities is from novel structures to structure-based drug discovery projects. Several high-impact MX publications have appeared from the two beamlines.

Worth noting from XRD1 beamline are the crystal structure of the lysenin pore (Podobnik *et al.*, 2016 ▸), the crystal structures of *Arabidopsis* and *Chlamydomonas* phospho­ribulokinase (Gurrieri *et al.*, 2019 ▸) and the structure of human *N*-acyl­phosphatidyl­ethano­lamine-hydrolyzing phospho­lipase D (Magotti *et al.*, 2015 ▸).

Highlights from the XRD2 beamline include a number of *de novo* structures, such as the complex between Uba4 (ubiquitin-like protein activator 4) and Urm1 (ubiquitin-related modifier 1), responsible for the synthesis of 2-thiol­ated wobble uridine (U_34_) in tRNA (Pabis *et al.*, 2020 ▸), the molecular architecture of the glycogen-committed PP1/PTG holoenzyme combining MX and SAXS analysis (Semrau *et al.*, 2022 ▸), and an integrated structural analysis of the C-terminal domain of the RTEL1 helicase (Cortone *et al.*, 2024 ▸).

A large part of the users’ community of XRD2 is involved in structure-based drug design projects, against targets such as carbonic anhydrase (implicated in glaucoma, epilepsy, cancer, and osteoporosis). An example is the structure of human carbonic anhydrase II in a complex with a carbonic anhydrase/telomerase dual-hybrid inhibitor (Berrino *et al.*, 2020 ▸). Among the inhibitors of other molecular targets, we wish to highlight the structure of thrombin with a G4-based aptamer with improved anti-coagulant properties (Troisi *et al.*, 2023 ▸), the structure of cathepsin K with a covalently bound alkyne inhibitor (Mons *et al.*, 2019 ▸), the inhibition of urease with mono- and di-substituted catechols (Mazzei *et al.*, 2021 ▸), and the molecular basis of the interaction between monoclonal antibodies and oligosaccharides involved in *Neisseria meningitidis* infections (Pietri *et al.*, 2024 ▸).

The Indo-Italian partnership for XRD2 has been productive, resulting in high impact publications, such as: the high resolution (∼1 Å) crystal structure of the complex of Fyn kinase SH3 domain with Tau protein, with implications in Alzheimer’s disease (Jos *et al.*, 2024 ▸); the crystal structure of the complex between DarT toxin and DarG antitoxin from *Mycobacterium tuberculosis* (Deep *et al.*, 2023 ▸); structural studies of neurotransmitter-inhibitor complexes implicated in pain and epilepsy therapies (Joseph *et al.*, 2022 ▸, Pidathala *et al.*, 2021 ▸); structure based drug design with SARS-COV2 (Panchariya *et al.*, 2021 ▸); and the crystal structure of the Y-family DNA polymerase from *Mycobacterium smegmatis* (Johnson *et al.*, 2019 ▸).

## 8. A vision for macromolecular crystallography at Elettra 2.0

In early 2023, Elettra conducted an informal survey to assess the current and future needs of its academic and industrial structural biology community. The results of the survey indicated that the majority of users opt for standard native MX data collection, especially for ligand screening, with some use of SAD phasing, and of advanced techniques like *in situ* plate screening, serial data collection and time-resolved MX. However, users anticipate a shift towards more diverse data collection methods in the future, with even greater interest in ligand screening, *in situ* plate screening and the integration of MX with spectroscopy.

At present, most users utilize a 50 µm × 50 µm beam, although there is a growing trend toward smaller 5 µm × 5 µm beams. Users have expressed interest in a κ-goniostat, as well as hardware and software for mesh scanning and fragment screening. Current microfocus beamlines, together with new crystal handling methods and the implementation of novel data processing approaches, allow one to obtain high resolution X-ray data also from crystals smaller than 5 µm. Such crystal size limits can be found with large macromolecular complexes, membrane proteins, or proteins with unstructured regions or viral particles, where the optimization of the crystallization kinetics and thermodynamics to increase the size of the crystals was not successful. In addition to traditional microcrystals obtained from purified proteins using standard batch crystallization techniques, a growing and very interesting area of application is the structural analysis of *in vivo* assembled viral superstructures (Coulibaly, 2019 ▸) or *in cellulo* crystallized recombinant protein (Schönherr *et al.*, 2024 ▸). To address these challenges and meet the future demands of structural biology, a new beamline for MX is being developed as part of the Elettra 2.0 upgrade to a fourth-generation light source. As part of this upgrade, which promises a significant increase in brightness (approximately 35 times at 1 keV and 160 times at 10 keV), the XRD2 beamline will be replaced with a new beamline.

**Figure 4:**
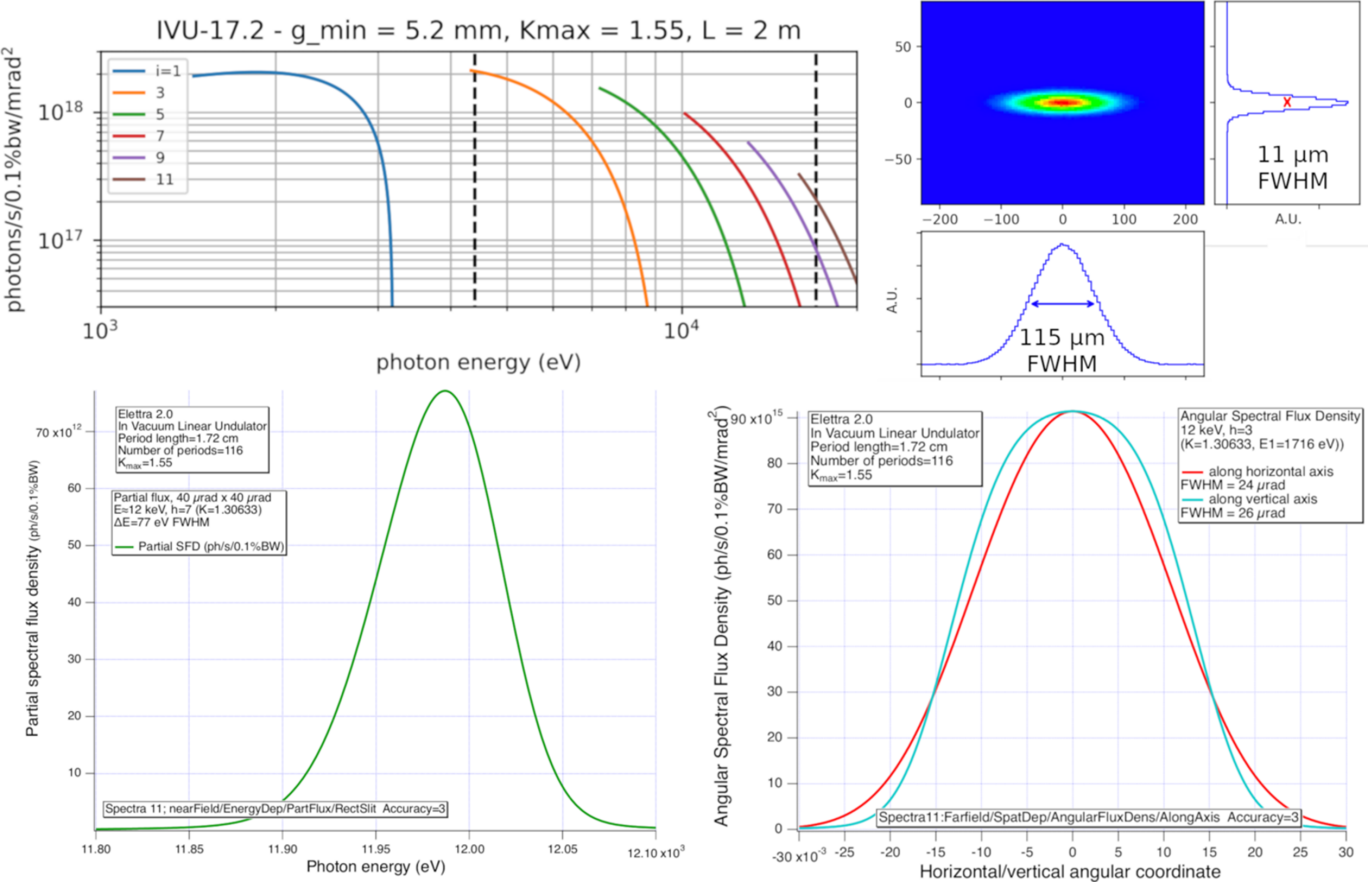
Expected performance of the µXRD in-vacuum undulator (period 17.2 mm, minimum gap 5.2 mm, 116 periods, *K*_max_ = 1.55, Elettra 2.0 parameters, 2.4 GeV, 400 mA). From top-left clockwise: spectral flux, showing odd harmonics; spatial photon beam size at 12 keV, *h* = 7; angular photon beam size at 12 keV, *h* = 7; partial 40 µrad × 40 µrad spectral flux density showing the energy line width at the monochromator.

**Figure 5:**
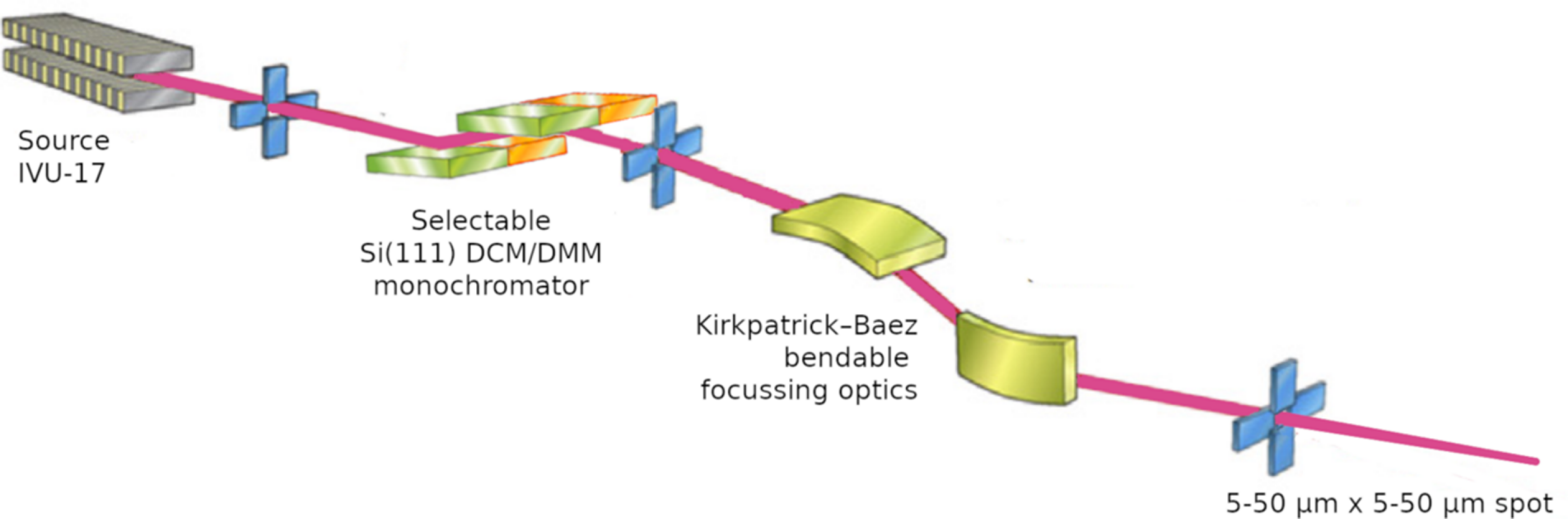
Optical scheme of the µXRD beamline at Elettra 2.0.

The new MX beamline, called µXRD, will be based on an in-vacuum undulator source, generating a low divergence, high brilliance beam (Fig. 4 ▸). The designed optical scheme (Fig. 5 ▸) foresees a cryogenic double crystal monochromator working in the range 5–17 keV and a focusing optical element based on a Kirkpatrick–Baez configuration. The monochromator will be able to work in a high resolution mode – based on Si(111) crystals – and in a high flux mode – based on a couple of multilayer mirrors having a bandwidth of ∼1% and expected to guarantee an increase of an order of magnitude in flux.

**Figure 6:**
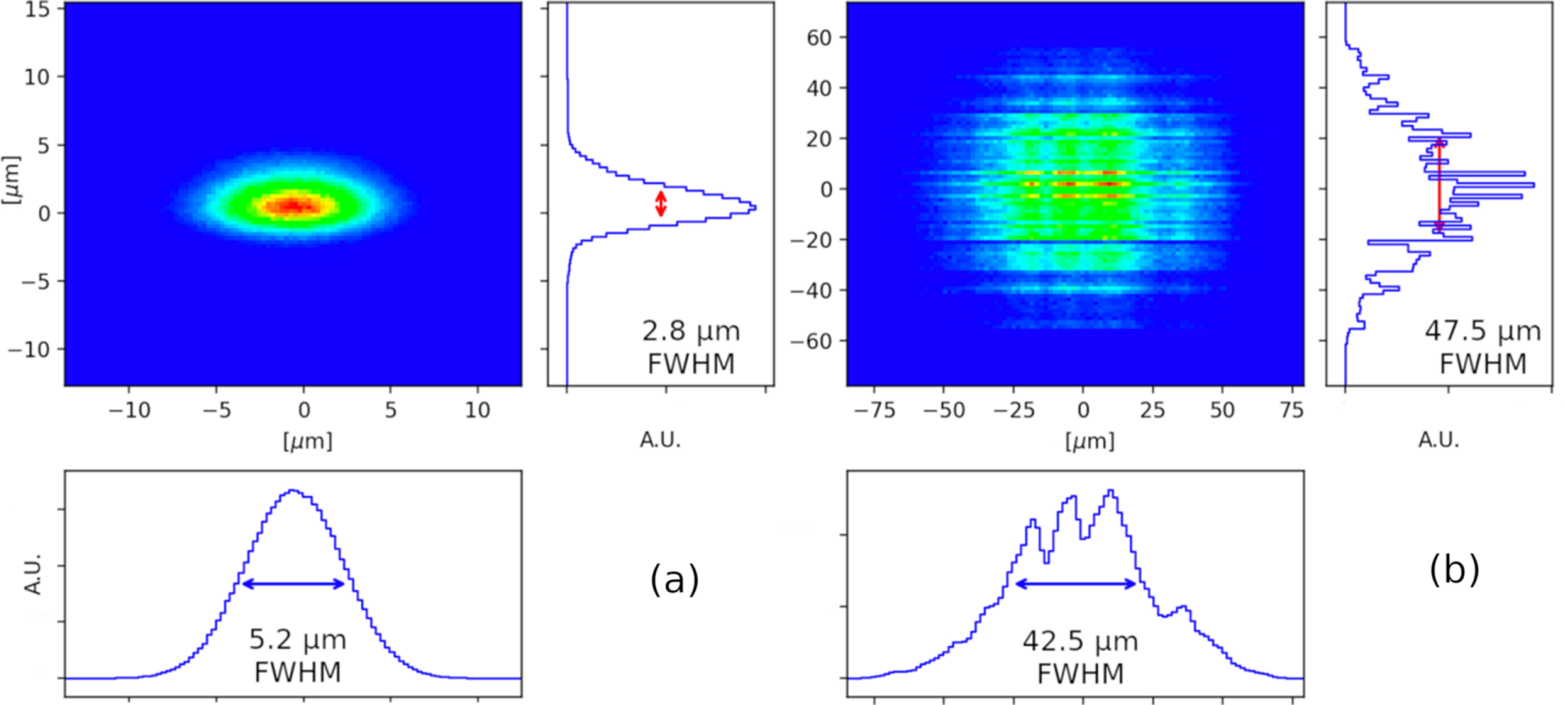
Ray tracing simulations based on Elettra 2.0 phase 2 machine parameters for the µXRD beam spot at the sample position using *DABAM/ShadowOui* simulated profiles (slope error of 0.15 µrad and 0.3 µrad for the vertical and horizontal focusing mirror, respectively) show the possibility to reach (*a*) the expected final beam size of 5 µm × 5 µm, expandable to (*b*) 50 µm × 50 µm.

The expected flux at the sample in high resolution mode is ∼5 × 10^12^ photons s^−1^ within a spot size of 5 µm × 5 µm (FWHM), quickly expandable up to 50 µm × 50 µm (Fig. 6 ▸) and beyond. The beamline will therefore be able to host small samples and offer the possibility to screen/scan larger ones, collect complete datasets faster, handle radiation damage better, and enable serial synchrotron crystallography experiments on fixed targets, including at room temperature. Together with the newly upgraded crystallization facility, it will provide a state-of-the-art platform for high-throughput crystallization and structure-based drug discovery. The planned experimental setup includes a fast, large-area detector, automounter and multi-axis goniometry, coupled with an infrastructure control software and pipelines for automated data collection, processing, evaluation and interpretation, aided by the incoming artificial intelligence. The µXRD beamline is due to come into operation in 2027.

## 9. Conclusions

Macromolecular X-ray crystallography represents a cornerstone of user operations at the Elettra Synchrotron, attracting a diverse community of researchers from across Europe and beyond. The XRD2 beamline, dedicated to MX measurements, provides a high photon flux and integrates advanced hardware and software automation within user-friendly, web-based interfaces.

As part of Elettra’s upgrade to a diffraction-limited machine, XRD2 will be replaced by µXRD, a next-generation beamline offering significantly enhanced brilliance. This advancement is set to unlock new possibilities for studying challenging samples and employing innovative data-collection methods.

To complement both current and future MX beamlines, Elettra has strategically implemented supporting facilities, including protein production and crystallization laboratories. These are being further strengthened with the addition of a state-of-the-art cryo-electron microscopy facility, ensuring a comprehensive infrastructure for structural biology research.

## Figures and Tables

**Table 1:** Source and optical layout details for XRD2 macromolecular crystallography beamline

Source	
Period length	64 mm
Peak field	3.5 T
Total number of poles	49
Pole sequence	1/4, −3/4, 1, −1,…, 1, −3/4, 1/4
Internal aperture	81 mm (H) × 10.7 mm (V)
Total power	18.3 kW (2 GeV, 400 mA)

Optics	
Collimating mirror	Cylindrical mirror with 50 nm Pt-coating
Radius	14 km
Distance from source	21200 mm
Incidence angle	3 mrad
Active length	1200 mm

Monochromator	
Distance from source	24100 mm
Energy range	7.5–36 keV
Energy resolution (Δ*E*/*E*)	1.4 × 10^−4^ [Si (111)]

Focusing mirror	
Tangential radius	8.9 km
Sagittal radius	50 mm
Distance from source	27000 mm
Incidence angle	3 mrad
Active length	1200 mm

## References

[bb1] Agirre, J., Atanasova, M., Bagdonas, H., Ballard, C. B., Baslé, A., Beilsten-Edmands, J., Borges, R. J., Brown, D. G., Burgos-Mármol, J. J., Berrisford, J. M., Bond, P. S., Caballero, I., Catapano, L., Chojnowski, G., Cook, A. G., Cowtan, K. D., Croll, T. I., Debreczeni, J. É., Devenish, N. E., Dodson, E. J., Drevon, T. R., Emsley, P., Evans, G., Evans, P. R., Fando, M., Foadi, J., Fuentes-Montero, L., Garman, E. F., Gerstel, M., Gildea, R. J., Hatti, K., Hekkelman, M. L., Heuser, P., Hoh, S. W., Hough, M. A., Jenkins, H. T., Jiménez, E., Joosten, R. P., Keegan, R. M., Keep, N., Krissinel, E. B., Kolenko, P., Kovalevskiy, O., Lamzin, V. S., Lawson, D. M., Lebedev, A. A., Leslie, A. G. W., Lohkamp, B., Long, F., Malý, M., McCoy, A. J., McNicholas, S. J., Medina, A., Millán, C., Murray, J. W., Murshudov, G. N., Nicholls, R. A., Noble, M. E. M., Oeffner, R., Pannu, N. S., Parkhurst, J. M., Pearce, N., Pereira, J., Perrakis, A., Powell, H. R., Read, R. J., Rigden, D. J., Rochira, W., Sammito, M., Sánchez Rodríguez, F., Sheldrick, G. M., Shelley, K. L., Simkovic, F., Simpkin, A. J., Skubak, P., Sobolev, E., Steiner, R. A., Stevenson, K., Tews, I., Thomas, J. M. H., Thorn, A., Valls, J. T., Uski, V., Usón, I., Vagin, A., Velankar, S., Vollmar, M., Walden, H., Waterman, D., Wilson, K. S., Winn, M. D., Winter, G., Wojdyr, M. & Yamashita, K. (2023). *Acta Cryst.* D**79**, 449–461.

[bb2] Albani, S., Costanzi, E., Hoang, G. L., Kuzikov, M., Frings, M., Ansari, N., Demitri, N., Nguyen, T. T., Rizzi, V., Schulz, J. B., Bolm, C., Zaliani, A., Carloni, P., Storici, P. & Rossetti, G. (2024). *J. Chem. Inf. Model.* **64**, 892–904. 10.1021/acs.jcim.3c01497 PMC10865365 38051605

[bb3] Berrino, E., Angeli, A., Zhdanov, D. D., Kiryukhina, A. P., Milaneschi, A., De Luca, A., Bozdag, M., Carradori, S., Selleri, S., Bartolucci, G., Peat, T. S., Ferraroni, M., Supuran, C. T. & Carta, F. (2020). *J. Med. Chem.* **63**, 7392–7409. 10.1021/acs.jmedchem.0c00636 PMC8154556 32463228

[bb4] Boavida, A., Napolitano, L. M., Santos, D., Cortone, G., Jegadesan, N. K., Onesti, S., Branzei, D. & Pisani, F. M. (2024). *EMBO Rep.* **25**, 876–901. 10.1038/s44319-023-00044-y PMC10897178 38177925

[bb5] Bottega, R., Ravera, S., Napolitano, L. M. R., Chiappetta, V., Zini, N., Crescenzi, B., Arniani, S., Faleschini, M., Cortone, G., Faletra, F., Medagli, B., Sirchia, F., Moretti, M., de Lange, J., Cappelli, E., Mecucci, C., Onesti, S., Pisani, F. M. & Savoia, A. (2021). *J. Cell. Physiol.* **236**, 5664–5675. 10.1002/jcp.30265 33432587

[bb6] Cortone, G., Graewert, M. A., Kanade, M., Longo, A., Hegde, R., González–Magaña, A., Chaves–Arquero, B., Blanco, F. J., Napolitano, L. M. R. & Onesti, S. (2024). *Protein Sci.* **33**, e5093. 10.1002/pro.5093 PMC11344278 39180489

[bb7] Costanzi, E., Kuzikov, M., Esposito, F., Albani, S., Demitri, N., Giabbai, B., Camasta, M., Tramontano, E., Rossetti, G., Zaliani, A. & Storici, P. (2021). *Int. J. Mol. Sci.* **22**, 11779. 10.3390/ijms222111779 PMC8583849 34769210

[bb8] Coulibaly, F. (2019). *Advances in Virus Research*, edited by F. A. Rey, pp. 275–335. Academic Press.

[bb9] Deep, A., Singh, L., Kaur, J., Velusamy, M., Bhardwaj, P., Singh, R. & Thakur, K. G. (2023). *Structure*, **31**, 780–789.e4. 10.1016/j.str.2023.04.008 37167974

[bb10] Delagenière, S., Brenchereau, P., Launer, L., Ashton, A. W., Leal, R., Veyrier, S., Gabadinho, J., Gordon, E. J., Jones, S. D., Levik, K. E., McSweeney, S. M., Monaco, S., Nanao, M., Spruce, D., Svensson, O., Walsh, M. A. & Leonard, G. A. (2011). *Bioinformatics*, **27**, 3186–3192. 10.1093/bioinformatics/btr535 21949273

[bb11] De March, M., Barrera-Vilarmau, S., Crespan, E., Mentegari, E., Merino, N., Gonzalez-Magaña, A., Romano-Moreno, M., Maga, G., Crehuet, R., Onesti, S., Blanco, F. J. & De Biasio, A. (2018). *Nucleic Acids Res.* **46**, 9816–9828. 10.1093/nar/gky723 PMC6182140 30102405

[bb12] De March, M., Merino, N., Barrera-Vilarmau, S., Crehuet, R., Onesti, S., Blanco, F. J. & De Biasio, A. (2017). *Nat. Commun.* **8**, 13935. 10.1038/ncomms13935 PMC5234079 28071730

[bb13] De Piante, E., D’Aria, F., Napolitano, L. M. R., Amato, J., Pirrello, S., Onesti, S. & Giancola, C. (2023). *Sci. Rep.* **13**, 12610. 10.1038/s41598-023-39675-5 PMC10400533 37537265

[bb14] Djinović Carugo, K., Helliwell, J. R., Stuhrmann, H. & Weiss, M. S. (2005). *J. Synchrotron Rad.* **12**, 410–419. 10.1107/S0909049504025762 15968116

[bb15] Fisher, S. J., Levik, K. E., Williams, M. A., Ashton, A. W. & McAuley, K. E. (2015). *J. Appl. Cryst.* **48**, 927–932. 10.1107/S1600576715004847 PMC4453979 26089766

[bb16] Franciosi, A. & Kiskinova, M. (2023). *Eur. Phys. J. Plus*, **138**, 79. 10.1140/epjp/s13360-023-03654-6 PMC9872737 36712550

[bb111] Gambitta, A. (2010). *Diamond Light Source Proc.* **1**, e23.

[bb17] Gonzalez-Magaña, A., Ibáñez de Opakua, A., Romano-Moreno, M., Murciano-Calles, J., Merino, N., Luque, I., Rojas, A. L., Onesti, S., Blanco, F. J. & De Biasio, A. (2019). *J. Biol. Chem.* **294**, 3947–3956. 10.1074/jbc.RA118.006391 PMC6422072 30655288

[bb18] Gossen, J., Albani, S., Hanke, A., Joseph, B. P., Bergh, C., Kuzikov, M., Costanzi, E., Manelfi, C., Storici, P., Gribbon, P., Beccari, A. R., Talarico, C., Spyrakis, F., Lindahl, E., Zaliani, A., Carloni, P., Wade, R. C., Musiani, F., Kokh, D. B. & Rossetti, G. (2021). *ACS Pharmacol. Transl. Sci.* **4**, 1079–1095. 10.1021/acsptsci.0c00215 PMC8009102 34136757

[bb19] Gregoratti, L., Lizzit, S., Karantzoulis, E. & Franciosi, A. (2024). *Synchrotron Radiat. News* **37**(1), 39–44.

[bb20] Gurrieri, L., Del Giudice, A., Demitri, N., Falini, G., Pavel, N. V., Zaffagnini, M., Polentarutti, M., Crozet, P., Marchand, C. H., Henri, J., Trost, P., Lemaire, S. D., Sparla, F. & Fermani, S. (2019). *Proc. Natl Acad. Sci. USA*, **116**, 8048–8053. 10.1073/pnas.1820639116 PMC6475412 30923119

[bb21] Johnson, M. K., Kottur, J. & Nair, D. T. (2019). *Nucleic Acids Res.* **47**, 10693–10705. 10.1093/nar/gkz792 PMC6846668 31544946

[bb22] Jos, S., Poulose, R., Kambaru, A., Gogoi, H., Dalavaikodihalli Nanjaiah, N., Padmanabhan, B., Mehta, B. & Padavattan, S. (2024). *J. Mol. Biol.* **436**, 168445. 10.1016/j.jmb.2024.168445 38218365

[bb23] Joseph, D., Nayak, S. R. & Penmatsa, A. (2022). *EMBO J.* **41**, e110735. 10.15252/embj.2022110735 PMC9340486 35796008

[bb24] Kabsch, W. (2010). *Acta Cryst.* D**66**, 125–132. 10.1107/S0907444909047337 PMC2815665 20124692

[bb25] Karantzoulis, E., Di Mitri, S., Barbo, F., Barletta, W., Bassanese, S., Bracco, R., Brajnik, G., Buonanno, A., Caiazza, D., Carniel, A., Castronovo, D., Cautero, M., Cleva, S., Comisso, M., Cudin, I., Dastan, S., De Monte, R., Diviacco, B., Fabris, A., Fabris, R., Gaio, G., Grulja, S., Gregoratti, L., Gubertini, A., Krecic, S., Lizzit, S., Loda, G., Lonza, M., Manukyan, K., Mazzucco, B., Milani, M., Millo, D., Modica, M., Novinec, L., Pangon, G., Pasotti, C., Passarelli, A., Rumiz, L., Sbarra, S., Scrimali, G., Shafqat, N., Simonetti, G., Svandrlik, M., Tripaldi, F., Veronese, M., Visintini, R., Yousefi, E. & Zaccaria, M. (2024). *Nucl. Instrum. Methods Phys. Res. A*, **1060**, 169007.

[bb26] Kuzikov, M., Costanzi, E., Reinshagen, J., Esposito, F., Vangeel, L., Wolf, M., Ellinger, B., Claussen, C., Geisslinger, G., Corona, A., Iaconis, D., Talarico, C., Manelfi, C., Cannalire, R., Rossetti, G., Gossen, J., Albani, S., Musiani, F., Herzog, K., Ye, Y., Giabbai, B., Demitri, N., Jochmans, D., Jonghe, S. D., Rymenants, J., Summa, V., Tramontano, E., Beccari, A. R., Leyssen, P., Storici, P., Neyts, J., Gribbon, P. & Zaliani, A. (2021). *Am. Chem. Soc. Pharmacol. Transl. Sci.* **4**, 1096–1110. 10.1021/acsptsci.0c00216 PMC7986981 35287429

[bb27] Lausi, A., Polentarutti, M., Onesti, S., Plaisier, J. R., Busetto, E., Bais, G., Barba, L., Cassetta, A., Campi, G., Lamba, D., Pifferi, A., Mande, S. C., Sarma, D. D., Sharma, S. M. & Paolucci, G. (2015). *Eur. Phys. J. Plus*, **130**, 43.

[bb28] Magotti, P., Bauer, I., Igarashi, M., Babagoli, M., Marotta, R., Piomelli, D. & Garau, G. (2015). *Structure*, **23**, 598–604. 10.1016/j.str.2014.12.018 PMC4351732 25684574

[bb29] Mazzei, L., Contaldo, U., Musiani, F., Cianci, M., Bagnolini, G., Roberti, M. & Ciurli, S. (2021). *Angew. Chem. Int. Ed.* **60**, 6029–6035. 10.1002/anie.202014706 33245574

[bb30] Mons, E., Jansen, I. D. C., Loboda, J., van Doodewaerd, B. R., Hermans, J., Verdoes, M., van Boeckel, C. A. A., van Veelen, P. A., Turk, B., Turk, D. & Ovaa, H. (2019). *J. Am. Chem. Soc.* **141**, 3507–3514. 10.1021/jacs.8b11027 PMC6396318 30689386

[bb31] Mueller, U., Thunnissen, M., Nan, J., Eguiraun, M., Bolmsten, F., Milàn-Otero, A., Guijarro, M., Oscarsson, M., de Sanctis, D. & Leonard, G. (2017). *Synchrotron Radiat. News* **30**(1), 22–27.

[bb32] Mueller-Dieckmann, C., Panjikar, S., Tucker, P. A. & Weiss, M. S. (2005). *Acta Cryst.* D**61**, 1263–1272. 10.1107/S0907444905021475 16131760

[bb33] Mueller-Dieckmann, C., Polentarutti, M., Djinovic Carugo, K., Panjikar, S., Tucker, P. A. & Weiss, M. S. (2004). *Acta Cryst.* D**60**, 28–38. 10.1107/s0907444903020833 14684889

[bb34] Owen, R. L., Rudiño-Piñera, E. & Garman, E. F. (2006). *Proc. Natl Acad. Sci. USA*, **103**, 4912–4917. 10.1073/pnas.0600973103 PMC1458769 16549763

[bb35] Pabis, M., Termathe, M., Ravichandran, K. E., Kienast, S. D., Krutyhołowa, R., Sokołowski, M., Jankowska, U., Grudnik, P., Leidel, S. A. & Glatt, S. (2020). *EMBO J.* **39**, e105087. 10.15252/embj.2020105087 PMC7527816 32901956

[bb36] Panchariya, L., Khan, W. A., Kuila, S., Sonkar, K., Sahoo, S., Ghoshal, A., Kumar, A., Verma, D. K., Hasan, A., Khan, M. A., Jain, N., Mohapatra, A. K., Das, S., Thakur, J. K., Maiti, S., Nanda, R. K., Halder, R., Sunil, S. & Arockiasamy, A. (2021). *Chem. Commun.* **57**, 10083–10086. 10.1039/d1cc03563k 34514483

[bb37] Pelliccia, S., Cerchia, C., Esposito, F., Cannalire, R., Corona, A., Costanzi, E., Kuzikov, M., Gribbon, P., Zaliani, A., Brindisi, M., Storici, P., Tramontano, E. & Summa, V. (2022). *Eur. J. Med. Chem.* **244**, 114853. 10.1016/j.ejmech.2022.114853 PMC9575579 36332546

[bb38] Pidathala, S., Mallela, A. K., Joseph, D. & Penmatsa, A. (2021). *Nat. Commun.* **12**, 2199. 10.1038/s41467-021-22385-9 PMC8044178 33850134

[bb39] Pietri, G. P., Bertuzzi, S., Karnicar, K., Unione, L., Lisnic, B., Malic, S., Miklic, K., Novak, M., Calloni, I., Santini, L., Usenik, A., Romano, M. R., Adamo, R., Jonjic, S., Turk, D., Jiménez-Barbero, J. & Lenac Rovis, T. (2024). *Carbohydr. Polym.* **341**, 122349. 10.1016/j.carbpol.2024.122349 38876728

[bb40] Podobnik, M., Savory, P., Rojko, N., Kisovec, M., Wood, N., Hambley, R., Pugh, J., Wallace, E. J., McNeill, L., Bruce, M., Liko, I., Allison, T. M., Mehmood, S., Yilmaz, N., Kobayashi, T., Gilbert, R. J. C., Robinson, C. V., Jayasinghe, L. & Anderluh, G. (2016). *Nat. Commun.* **7**, 11598. 10.1038/ncomms11598 PMC4865846 27176125

[bb41] Polentarutti, M., Glazer, R. & Djinović Carugo, K. (2004). *J. Appl. Cryst.* **37**, 319–324.

[bb42] Schönherr, R., Boger, J., Lahey-Rudolph, J. M., Harms, M., Kaiser, J., Nachtschatt, S., Wobbe, M., Duden, R., König, P., Bourenkov, G., Schneider, T. R. & Redecke, L. (2024). *Nat. Commun.* **15**, 1709. 10.1038/s41467-024-45985-7 PMC10894269 38402242

[bb43] Semrau, M. S., Giachin, G., Covaceuszach, S., Cassetta, A., Demitri, N., Storici, P. & Lolli, G. (2022). *Nat. Commun.* **13**, 6199. 10.1038/s41467-022-33693-z PMC9582199 36261419

[bb44] Stefanelli, I., Corona, A., Cerchia, C., Cassese, E., Improta, S., Costanzi, E., Pelliccia, S., Morasso, S., Esposito, F., Paulis, A., Scognamiglio, S., Di Leva, F. S., Storici, P., Brindisi, M., Tramontano, E., Cannalire, R. & Summa, V. (2023). *Eur. J. Med. Chem.* **253**, 115311. 10.1016/j.ejmech.2023.115311 PMC10068823 37043904

[bb45] Troisi, R., Napolitano, V., Rossitto, E., Osman, W., Nagano, M., Wakui, K., Popowicz, G. M., Yoshimoto, K. & Sica, F. (2023). *Nucleic Acids Res.* **51**, 8880–8890. 10.1093/nar/gkad634 PMC10484730 37503836

[bb46] Vonrhein, C., Flensburg, C., Keller, P., Sharff, A., Smart, O., Paciorek, W., Womack, T. & Bricogne, G. (2011). *Acta Cryst.* D**67**, 293–302. 10.1107/S0907444911007773 PMC3069744 21460447

[bb47] Weiss, M. S., Mander, G., Hedderich, R., Diederichs, K., Ermler, U. & Warkentin, E. (2004). *Acta Cryst.* D**60**, 686–695. 10.1107/S0907444904003002 15039557

[bb48] Winter, G. & McAuley, K. E. (2011). *Methods*, **55**, 81–93. 10.1016/j.ymeth.2011.06.010 21763424

[bb49] Zeldin, O. B., Gerstel, M. & Garman, E. F. (2013). *J. Appl. Cryst.* **46**, 1225–1230.

[bb50] Zhang, Z., Sauter, N. K., van den Bedem, H., Snell, G. & Deacon, A. M. (2006). *J. Appl. Cryst.* **39**, 112–119.

