# Macromolecular crystallography at Elettra: current and future perspectives. Corrigendum

**DOI:** 10.1107/S1600577526003206

**Published:** 2026-03-30

**Authors:** Raghurama P. Hegde, Nicola Demitri, Annie Héroux, Alessandro Olivo, Giorgio Bais, Michele Cianci, Paola Storici, Dan-George Dumitrescu, Nishant Kumar Varshney, Balasubramanian Gopal, D. D. Sarma, Lisa Vaccari, Silvia Onesti, Maurizio Polentarutti

**Affiliations:** a Elettra – Sincrotrone Trieste SCpA SS 14 km 163,5 in AREA Science Park Basovizza 34149Trieste Italy; b Former Elettra – Sincrotrone Trieste SCpA SS 14 km 163,5 in AREA Science Park Basovizza 34149Trieste Italy; c Department of Agricultural, Food and Environmental Sciences, Università Politecnica delle Marche, via Brecce Bianche 10, 60131Ancona, Italy; d Nobian, Van Asch v. Wijckstraat 53, 3811 LPAmersfoort, The Netherlands; e IR Technology Services Pvt Ltd, EL-91, TTC Industrial Area, Electronic Zone, Mahape, Navi Mumbai, Maharashtra400710, India; f Molecular Biophysics Unit, Division of Biological Sciences, Indian Institute of Science, Bengaluru560012, India; g Solid State and Structural Chemistry Unit, Indian Institute of Science, Bengaluru560012, India; Cornell University, USA

**Keywords:** data collection, X-ray diffraction, macromolecular crystallography, Elettra upgrade

## Abstract

Corrigendum to the article by Hegde *et al.* [(2025). *J. Synchrotron Rad.***32**, 757–765].

An institutional oversight made in the description of the collaboration relating to the CryoEM facility mentioned in the article by Hegde *et al.* (2025 ▸) is hereby corrected by the following statement:

The cryo-EM facility implemented through the PRP@CERIC project (https://www.pathogen-ri.eu/), coordinated by Area Science Park and funded by the European Union through the National Recovery and Resilience Plan (NRRP), will be jointly managed by the CNR Istituto Officina dei Materiali (CNR-IOM) and by the CNR Istituto di Cristallografia (CNR-IC), in collaboration with Elettra Sincrotrone Trieste. In this connection, wherever CNR-IOM was mentioned in the text, CNR-IC should have been mentioned as well.
